# Human Temperaments. XII.—The Philosopher


**Published:** 1916-10-07

**Authors:** 


### HUMAN TEMPERAMENTS.

### XII. The Philosopher.*

"You are a philosopher, Dr. Johnson. I have tried too in my time to be a philosopher; but, I don't know how, cheerfulness was always breaking in." This notion of the philosopher as a scowling, morose, gloomy person was never true, and was certainly not long prevalent, but it seems, from Mr. Edwards' confession to Dr. Johnson, quoted above, that it prevailed in the eighteenth century. Mr. Edwards should have remembered that Democritus was called the Laughing Philosopher, and if ever the philosopher was regarded as by nature a pessimist, that time is long past, even although the character of Carlyle gave some countenance to it. We do not now regard the philosopher as a growling, scowling pessimist, but we do regard him as a person who is not put out about little things; who does not storm when his dinner is late, or, like Carlyle, call down fire from heaven when he cannot lay his hand upon the match-box. Experience scarcely bears out this opinion. The philosopher has his full share of the infirmities of human nature. Even Dr. Johnson himself was not indifferent to the composition of his dinner, and in the controversy between him and Adam Smith both of them said things much better left unsaid. In respect of their reactions to agreement and opposition, philosophers are much like other men. Agree with their peculiar doctrines and they will coo like doves and purr like kittens; but cast a doubt on their impeccability and the coo of the dove is apt to change into the scream of the angry parrot, and the kitten bares his teeth and shows his claws.

### The Antithesis of the Practical Man.

No. The philosopher is distinguished, not by the equability of his temper, but by his devotion to thought rather than action, to principles rather than details. He searches for rules. He is a generaliser. To him an isolated fact has no value; indeed, he scarcely recognises that there can be an isolated fact. A fact is to him of value only as far as it illustrates, corroborates, or contradicts a principle, or is explained by a principle, or needs a new principle to explain it. He is the antithesis of the practical man, who despises general principles, calls the generaliser a theoretical fool, and stores his memory with disconnected facts; and he is the complement of the man of action, whose function is to apply general principles to particular cases, and especially of the inventor, whose function is to find new applications for general principles.

With action the philosopher as such has nothing to do. His business is to discover general truths, and though the discovery of a general truth always has, sooner or later, a bearing upon action, yet its bearing upon action is not often immediate, and may be very remote. Moreover, the bearing of a general truth on action, and its application to concrete cases, is rarely apparent at the time of its discovery. No philosopher ever sets out deliberately to discover what shall be of practical use and application. He sets out to discover truth; and the practical application comes afterwards, it may be a long time afterwards, and is seldom made by the philosopher himself. Neither Copernicus nor Galileo had any expectation that the discovery of the rotation of the earth would be applied to navigation, and enable the course of a ship to be as accurately laid and kept as the course of a land journey. Clerk-Maxwell little thought that his recondite mathematical speculations would render wireless telegraphy possible. Faraday pursued his researches into electricity and magnetism without a thought of the electric telegraph and the electric motor in which they resulted. Mendel pursued his researches into inheritance with no view to the improvements in methods of breeding to which they have directly led. Pasteur never dreamed that his researches into the action of yeast would lead to the abolition of sepsis in wounds and the sensible prolongation of human life.

### An Attempt Foredoomed to Failure.

It would be scarcely too much to say that those investigations which have been made with a direct view to utility have been the least useful. It is so at least with investigations into the incidence of the weather. A strong but rather confused recognition of these truths leads the philosopher to look askance upon researches that have a directly utilitarian object, a feeling that finds expression in the toast reputed to have been proposed at a dinner of the Royal Society: "Here's to the latest scientific discovery, and may it never be of the slightest use to anyone!" It would be vain for philosophers to attempt to exclude usefulness from their discoveries, even if the attempt were to be seriously made. We can never foresee in what unexpected ways a recondite and apparently fanciful discovery may prove useful. But though the attempt is never seriously made, and must be unsuccessful if it were made, yet it is most desirable that investigations should never be discouraged on the ground that their utility is not apparent.

It has been said that everyone is from his birth either a Platonian or an Aristotelian. The universality of the saying is to seek, but if for everyone we substitute every philosopher, and if for a Platonian or an Aristotelian we substitute a man of deductive or of inductive mind, which is approximately what the saying means, we shall not be far astray. It is thus that men of philosophic temperament may be divided. The man of deductive mind revels in abstractions. He formulates in the widest terms a general principle, and proceeds to deduce from it subsidiary principles, until he has erected a comprehensive and coherent system, whose principles explain all the facts within its ambit. The inductive mind begins by collecting

* The previous articles appeared on May 6, 13, 27, June 10 and 24, July 8 and 22, Aug. 5 and 19, and Sept. 9 and 23, 1916.

and examining facts. After a sufficient number have been examined, their resemblances and differences noted, there seems to be a principle running through them. This principle the inductive philosopher is quick to seize; but he does not yet build a system upon it. He looks round for relevant facts, and as he finds them he applies his principle to them and notes whether they sustain it. If they do, well and good. So far he is justified; but he does not stop here, he looks round for more. If amongst them all he finds a single one that is irreconcilable with his principle, the principle must go. It is abandoned at once and without hesitation, and another is substituted and submitted to the same ordeal, to be perhaps abandoned in its turn. Not until every relevant fact that he can find is shown to be consistent with his principle is that principle accepted.

Some Types and their Caricatures.

Each of these kinds of mind has its value, and each is the complement of the other. The deducer finds it easy to frame an hypothesis that will explain the facts, but he is apt to take insufficient account of the facts, and to forget or ignore facts that conflict with his principle. The inducer is apt to become entangled and confused in a mass of facts for which he can find no explanatory principle. He is apt to want the wide comprehensive grasp of his facts and the penetrating insight that discovers a principle running through them. The caricature of the deducer is the architect who designs a three-storeyed building without stairs, whose rooms are inaccessible, and whose windows give no light: the caricature of the inducer is the labourer who piles up a heap of bricks and calls it a building. Pythagoras, who explained everything by the principle of number, is the type of the crazy deducer; the mere collector, who compiles statistical tables, is the type of the crazy inducer. In Kepler and Darwin both the inductive and the deductive turn of mind were well developed, with a decided preponderance of the inductive: in Newton and Bentham they were both well developed, with a decided preponderance of deduction.

The inducer, with his keen appreciation of the value of fact, finding that facts seldom present themselves in Nature sufficiently pure and free from ambiguity for his purposes, usually devises means of isolating his facts. In other words, he is an experimenter. The deducer, perhaps because of his defective appreciation of the value of facts, is rarely an experimenter. When he does apply his principles to practical purposes his applications border on the ludicrous. Kant, who elaborated the Categorical Imperative, invented suspenders for his stockings to take the place of garters; Herbert Spencer, the formulator of the theory of evolution, invented ear-plugs to keep out trivial conversation. All the great inductive philosophers have been experimenters: none of the deductive philosophers has been an experimenter. The deducer looks upon his theories as his children, and resents any inconvenient fact that tends to injure them. He is apt first to resent and then to ignore it; and at length he becomes able to ignore immense classes of facts. Bentham and his followers of the utilitarian school steadily refused to admit that men ought to be actuated by any motive but that of material interest, and at length succeeded in convincing themselves that men are not actuated by any other motive; and this they held in spite of the whole evidence of history, which shows that for one war that has been waged for material gain two have been waged for religion, and two more for some other ideal.

The Deductive Philosopher.

Hegel is another conspicuous example of the crazy deducer. His philosophy may be put briefly thus: Everything is nothing, and nothing may be anything, must be something, and perhaps is everything. The purely deductive philosopher is a moonraker, and his native place is Gotham; but this must not blind us to the truth that inductive philosophy owes all its value to the deductive element that it contains. In other words, it is only by imagination, by speculation, that principles can be devised; and heaps of facts unvitalised by a principle are not knowledge: they are only the material out of which knowledge can be constructed, and by themselves are worthless. Speculation and fact are to each other as male and female, as sperm and germ. Each by itself is barren; but unite them, and the offspring is knowledge.

